# Poor oral health and risks of total and site-specific cancers in China: A prospective cohort study of 0.5 million adults

**DOI:** 10.1016/j.eclinm.2022.101330

**Published:** 2022-03-05

**Authors:** Xi Zhang, Ben Liu, Henry S Lynn, Kexin Chen, Hongji Dai

**Affiliations:** a Clinical Research Unit, Xin Hua Hospital, Shanghai Jiao Tong University School of Medicine, Shanghai, China; b Department of Epidemiology and Biostatistics, Tianjin Medical University Cancer Institute and Hospital, National Clinical Research Center for Cancer, Tianjin Key Laboratory of Cancer Prevention and Therapy, Tianjin's Clinical Research Center for Cancer, Key Laboratory of Molecular Cancer Epidemiology of Tianjin, Huanhuxi Road, Hexi District, Tianjin 300060, China; c Department of Biostatistics, School of Public Health, Fudan University, Shanghai, China

**Keywords:** Oral health, Oral hygiene, Gum bleeding, Tooth brushing, Gastronintestinal cancer, Cohort study

## Abstract

**Background:**

There is a strong connection between oral health and overall wellness. We aim to examine the association between poor oral health and the risk of developing or dying of cancer, and whether the association differs by residential area.

**Methods:**

Between 2004 and 2008, a total of 510,148 adults free of cancer were included from the China Kadoorie Biobank study and thereafter followed up to 2015. Poor oral health was assessed from a self-reported baseline questionnaire and defined as a combination of rarely brushing teeth and always gum bleeding. We used Cox proportional hazards models to estimate the hazard ratio (HR) of cancer risk and its associated 95% confidence interval (CI) according to oral health status.

**Findings:**

Overall, 14.9% of participants (19.7% in rural areas and 8.8% in urban areas) reported poor oral health at baseline. After 4,602,743 person-years of follow-up, we identified 23,805 new cancer cases and 11,973 cancer deaths, respectively. Poor oral health was associated with higher risks of total cancer incidence (HR: 1.08, 95% CI: 1.04–1.12) and death (HR: 1.10, 95% CI: 1.05–1.16). For the site-specific cancers, poor oral health was significantly associated with higher risk of stomach cancer incidence (cases: 2964, HR: 1.10, 95% CI: 1.00–1.22), esophageal cancer incidence (cases: 2119, HR: 1.19, 95% CI: 1.07–1.33), esophageal cancer death (cases: 1238, HR: 1.29, 95% CI: 1.12–1.49), liver cancer incidence (cases: 2565, HR: 1.18, 95% CI: 1.06–1.32), and liver cancer death (cases: 1826, HR: 1.20, 95% CI: 1.05–1.36). This positive association was stronger among rural residents compared to urban residents (interaction test *P* < 0.01).

**Interpretation:**

Our findings indicate that poor oral health is associated with higher risk for cancers, especially digestive system cancers. Promotion of oral health in the general population, especially for rural residents, could have valuable public health significance in preventing major systemic diseases.

**Funding:**

Supported by grants (2021YFC2500400, 2016YFC0900500, 2016YFC0900501, 2016YFC0900504) from the National Key Research and Development Program of China, grants from the Kadoorie Charitable Foundation in Hong Kong and grants grants (088158/Z/09/Z, 104085/Z/14/Z, 202922/Z/16/Z) from Wellcome Trust in the UK. CKB is supported by the Kadoorie Charitable Foundation (KCF) in Hong Kong.

**Research in context**

**Evidence before this study**

We searched PubMed, OVID, and China National Knowledge Infrastructure for studies published in English and Chinese, with no restrictions on publication date. We used the search terms “oral health”, or “oral hygiene”, or “toothbrushing”, or “gum bleeding” and “cancer”, or “neoplasms”, and found little reliable prospective evidence available in non-white populations about the associations of oral health with cancer risks.

**Added value of this study**

In a large population-based cohort including 510,148 Chinese general participants from urban and rural who were free of cancer at enrolment, we found nearly 10% higher risks for both total cancer incidence and death among participants who rarely/never brushed teeth versus those with normal oral health. This positive association was stronger among rural residents compared to urban residents. We also found that participants who rarely or never brushed teeth had a higher risk of stomach and esophageal cancer, while those with gum bleeding had a higher risk of liver cancer.

**Implications of all the available evidence**

Poor oral health is associated with higher risks of both cancer incidence and mortality. Moreover, poor oral health can have different impacts on different types of cancer. Promotion of oral health in the general population, especially for rural residents, may help reduce the risk of cancer.

Alt-text: Unlabelled box

## Introduction

Poor oral health is a major public health problem affecting over 3.5 billion people across the world.^1^ Dental health conditions can affect not only the oral cavity but also a person's general quality of life and the ability to eat and speak. There is a strong connection between oral health and overall wellness. In many low-income and middle-income areas, oral diseases remain a neglected and largely untreated issue because treatment costs exceed available resources.^2^ According to the Fourth National Oral Health Survey of China, almost 90% of Chinese adults suffered from periodontal disease of various severities.^3^ However, the knowledge, attitudes and practices of periodontal health are generally poor, with less than 20% of Chinese adults being knowledgeable about periodontal disease, especially in rural residents.^4^

Common oral diseases and conditions include dental cavities, gingivitis and periodontal disease. Poor oral hygiene, such as not brushing teeth properly or regularly, is a leading contributor to oral diseases. Oral diseases or inadequate oral hygiene is associated with a variety of serious health issues, such as cardiovascular diseases (CVD),^5^, ^6^, ^7^, ^8^ diabetes,^9^ liver diseases,^10^ pulmonary disease,^11^ and cancer,^12^ although few large-scale epidemiologic studies on periodontal disease and incident cancer exist.^13^ Recently, the China Kadoorie Biobank (CKB) cohort of 0.5 million participants has provided new evidence that less frequent toothbrushing is associated with a higher risk for vascular events and nonvascular disease such as cancer.^5^ However, because cancer is a heterogeneous disease, poor oral health can have different impacts on different types of cancer. Also, previous studies conducted in western countries have reported strong relationships between gum disease and pancreatic^14^^,^^15^ and oropharyngeal cancer,^16^ but these results may not be generalizable to non-white populations given the cancer disparity between different population groups. For example, periodontitis and edentulism were not associated with cancer risk among black adults.^17^ In short, few prospective non-white cohorts have comprehensively examined site-specific cancers.

In the present study, we assess the association between oral health status and risks of total and site-specific cancers based on a large prospective cohort of Chinese adults. In addition, we explore potential effect modification by residential area.

## Methods

### Study population

CKB is a prospective cohort study of 512,726 adults aged 30–79 years from ten geographically diverse areas across China. Details of its study design have been described elsewhere.^18^, ^19^, ^20^ Briefly, trained health workers administered a laptop-based questionnaire on sociodemographic characteristics, dietary and lifestyle factors, personal health and medical histories, and measured anthropometric indexes at baseline. Participants were enrolled between 2004 and 2008, and have been followed up subsequently for assessment of morbidities and mortality. The study protocol was approved by the ethics review committee of the Chinese Centers for Disease Control and Prevention (Beijing, China) and the Oxford Tropical Research Ethics Committee, University of Oxford (Oxford, United Kingdom). Institutional review boards at all participating centers approved the study. All participants gave informed consent before taking part in the study.^19^ This study followed the Strengthening the Reporting of Observational Studies in Epidemiology (STROBE) reporting guidelines.

In this investigation, we excluded 2578 patients who reported a medical history of cancer at baseline, and performed the final analyses on 209,236 men and 300,912 women. Analyses used CKB data release 17.02.

### Assessment of oral health status and other baseline characteristics

Baseline oral health status was determined by the question “How often do your gums bleed when you brush your teeth?” with four mutually exclusive answers: (a) occasionally, rarely or never; (b) sometimes; (c) always; (d) rarely or never brush teeth. Individual with gum bleeding occasionally or sometimes may have incorrect brushing technique or brush teeth too vigorously. Therefore, we consider answers a or b as an indicator of normal oral health and answers c or d as an indicator of poor oral health. To test the reproducibility of the answers, we included 1315 participants who completed the same questionnaire twice at an interval of less than 1.5 years, and got an acceptable internal consistency (Kappa = 0.598, **Supplemental Table S1**).

Information on demographics, dietary intake, physical activity, weight, height, smoking and alcohol drinking habits, family history of cancer, and other exposures was collected from the baseline questionnaire. Body weight and height were measured by trained staff using calibrated instruments. Body mass index (BMI) was estimated as weight in kilograms divided by height in meters squared. Self-reported smoking status was validated using an exhaled carbon monoxide test.^21^ Self-reported alcohol consumption was estimated as grams of pure alcohol per week based on beverage type, amount consumed per occasion and drinking frequency.^22^ Physical activity levels were estimated by multiplying the energy expenditure in metabolic equivalent tasks (MET) measured in hours per day of each activity by hours spent on the activity and summing the values of all activities in MET hours a day (MET hr/day).^23^

### Follow-up and outcome measures

Long-term follow-up for outcome data was done through active follow-up, as well as by electronic linkage to three systems: (1) mortality registries in each study region; (2) morbidity registries for cancer in each study region; and (3) the new nationwide health insurance system covering about 98% of the CKB participants. Detailed information about each hospital admission, including dates of admission and discharge, description and International Classification of Disease 10th Revision (ICD-10) code of the condition, and detailed procedure codes were collected and processed.^19^ By December 31, 2015, 37,881 participants (7.4%) had died, and only 3903 (0.76%) were lost to follow up in the CKB cohort.

The primary outcomes of this study were incidence and death from any cancer (ICD-10: C00-C97). We further analyzed the 10 most common cancer types in the CKB cohort,^24^ including lung (C33-C34), female breast (C50), stomach (C16), esophageal (C15), liver (C22), colorectal (C18-C20), cervis uteri (C53), pancreas (C25), head and neck (C00-C14), leukemia and lymphoma (C91-C95, C81-C85).

### Statistical analysis

Person-years were calculated from the date of entry into the cohort until the date of first cancer diagnosis (for incidence), death due to cancer (for mortality), death due to other causes, lost to follow-up (for censoring), or the end of the observation period at 31 December 2015, whichever came first. We used unadjusted and adjusted Cox proportional hazard regression models to estimate hazard ratios (HRs) and their 95% confidence intervals (CIs) for cancer risk according to oral health status (i.e. rarely brushing teeth, gum always bleeding, or both). Covariates in the adjusted models included age (continuous), sex (male or female), BMI (continuous), study site (10 geographical areas), education level (no formal school, primary or middle school, or high school and above), marital status (married or other), household income per year (< ¥10,000, ¥10,000–19,999, ¥20,000–34,999, or ≥ ¥35,000), alcohol consumption (non-drinker, occasional drinker, former drinker, or regular drinker), smoking status (never smoker, occasional smoker, former smoker, or regular smoker), physical activity in MET hr/day (continuous), aspirin prescription for CVD (no, yes, or missing), menopausal status (for women only, premenopausal, perimenopausal, or postmenopausal), personal history of diabetes (no or yes), and family history of cancer (no or yes). Schoenfeld's goodness-of-fit test was used to assess the proportional hazards assumption for the Cox regression models.^25^ Age-adjusted incidence and survival curves were presented to compare the cumulative cancer incidence or mortality between the normal oral health group and the poor oral health group.

We further examined the associations of oral health status with total cancer incidence or mortality among pre-specified baseline subgroups based on age, sex, menopausal status, BMI, study region, annual household income, smoking status, alcohol drinking, aspirin prescription, history of diabetes and family history of cancer, adjusted for similar covariates. We also carried out analyses for site-specific cancers and examined potential effect modification by residential status (rural versus urban). Tests for interaction were performed using the Wald test for the cross product interaction term. Moreover, to examine the robustness of our findings, we performed sensitivity analyses by a) excluding participants who developed cancer (*N* = 7730) or died from cancer (*N* = 5236) during the first 2 years of follow-up; and b) conducting competing risk regression analyses using the method proposed by Fine and Gray,^26^ where death from other causes was considered as a competing event.

All statistical analyses were conducted using the SAS software (version 9.4; SAS Institute, Cary, NC, USA) and Stata (version 14; StataCorp, College Station, TX, USA). All statistical tests were two-tailed, and the significance level was set at the 5% level.

### Role of funding sources

The funders of this study had no role in study design, data collection, data analysis, data interpretation, or writing of the manuscript. This manuscript was not prepared in collaboration with investigators of the CKB and does not necessarily reflect the opinions or views of the CKB, the KCF, or the institutions participating in the CKB.

## Results

### Participants characteristics

The present study included 510,148 participants with a median age of 51 years at baseline (Table 1). Overall, 14.9% of the participants reported having poor oral health (5.3% always gum bleeding, 9.5% rarely or never brushed teeth). Compared to participants with normal oral health, participants who rarely or never brushed their teeth were more likely to be older, males, rural residents, less educated, with low income, with low BMI, smokers or alcohol drinkers, and postmenopausal if they were females. Baseline characteristics were similar between participants with their gums always bleeding and those with normal oral health.

**Table 1:** Baseline characteristics of study participants by oral health status in the CKB study.

Characteristics	Overall	Normal	Gum bleeding	Rarely or never brush teeth
**Participant, N**	510,148	434,254	27,218	48,676
**Age (year), median (range)**	51 (30–80)	51 (30–80)	47 (30–78)	60 (30–80)
**Sex, N (%)**				
**Men**	209,236 (41.01)	176,849 (40.72)	7831 (28.77)	24,556 (50.45)
**Women**	300,912 (58.99)	257,405 (59.28)	19,387 (71.23)	24,120 (49.55)
**Baseline BMI (mean ± SD, kg/m^2^)**	23.66±3.38	23.7 ± 3.36	23.75±3.31	23.21±3.58
**Study site**^a^				
**Urban**	224,769 (44.06)	205,000 (47.21)	14,062 (51.66)	5707 (11.72)
**Rural**	285,379 (55.94)	229,254 (52.79)	13,156 (48.34)	42,969 (88.28)
**Marital status**				
**Currently married**	462,219 (90.6)	396,437 (91.29)	25,295 (92.93)	40,487 (83.18)
**Other**	47,929 (9.40)	37,817 (8.71)	1923 (7.07)	8189 (16.82)
**Educational levels**				
**No formal school**	94,729 (18.57)	73,026 (16.82)	4737 (17.40)	16,966 (34.85)
**Primary or middle school**	164,277 (32.20)	138,000 (31.78)	7536 (27.69)	18,741 (38.50)
**High school and above**	251,142 (49.23)	223,228 (51.40)	14,945 (54.91)	12,969 (26.64)
**Household income, ¥ per year**				
**< ¥10,000**	143,993 (28.23)	112,159 (25.83)	7392 (27.16)	24,442 (50.21)
**¥10,000–19,999**	148,179 (29.05)	125,135 (28.82)	7590 (27.89)	15,454 (31.75)
**¥20,000–34,999**	126,078 (24.71)	113,120 (26.05)	6966 (25.59)	5992 (12.31)
**≥ ¥35,000**	91,898 (18.01)	83,840 (19.31)	5270 (19.36)	2788 (5.73)
**Physical activity (MET hr/day)**^b^	17.53 (10.38, 30.08)	17.8 (10.67, 30.24)	20.7 (12.22, 32.67)	13.18 (8.4, 25.26)
**Smoking status**				
**Never smoker**	315,830 (62.01)	270,300 (62.34)	20,081 (73.90)	25,449 (52.36)
**Former smoker**	42,909 (8.42)	35,219 (8.12)	2177 (8.01)	5513 (11.34)
**Occasional smoker**	22,815 (4.48)	18,869 (4.35)	1376 (5.06)	2570 (5.29)
**Regular smoker**	127,785 (25.09)	109,170 (25.18)	3539 (13.02)	15,076 (31.02)
**Alcohol drinking**				
**Non-drinker**	233,761 (45.82)	203,298 (46.82)	12,599 (46.29)	17,864 (36.70)
**Former drinker**	9083 (1.78)	7928 (1.83)	308 (1.13)	847 (1.74)
**Occasional drinker**	174,008 (34.11)	142,157 (32.74)	9725 (35.73)	22,126 (45.46)
**Regular drinker**	93,296 (18.29)	80,871 (18.62)	4586 (16.85)	7839 (16.10)
**Red meat**				
**Rarely or never**	149,196 (29.25)	136,211 (31.37)	9292 (34.14)	3693 (7.59)
**<1 day/month**	91,476 (17.93)	83,214 (19.16)	4581 (16.83)	3681 (7.56)
**1–3 days/week**	181,266 (35.53)	154,681 (35.62)	9677 (35.55)	16,908 (34.74)
**4–6 days/week**	63,861 (12.52)	45,372 (10.45)	2600 (9.55)	15,889 (32.64)
**Daily**	24,349 (4.77)	14,776 (3.40)	1068 (3.92)	8505 (17.47)
**Vegetable**				
**Rarely or never**	483,463 (94.77)	412,537 (95.00)	25,933 (95.28)	44,993 (92.43)
**<1 day/month**	18,004 (3.53)	14,886 (3.43)	880 (3.23)	2238 (4.60)
**1–3 days/week**	7161 (1.40)	5829 (1.34)	334 (1.23)	998 (2.05)
**4–6 days/week**	1393 (0.27)	905 (0.21)	66 (0.24)	422 (0.87)
**Daily**	127 (0.02)	97 (0.02)	5 (0.02)	25 (0.05)
**Fruit**				
**Rarely or never**	95,880 (18.79)	87,175 (20.07)	6136 (22.54)	2569 (5.28)
**<1 day/month**	47,746 (9.36)	43,146 (9.94)	2801 (10.29)	1799 (3.70)
**1–3 days/week**	160,630 (31.49)	142,838 (32.89)	8866 (32.57)	8926 (18.34)
**4–6 days/week**	173,310 (33.97)	138,506 (31.90)	7872 (28.92)	26,932 (55.33)
**Daily**	32,582 (6.39)	22,589 (5.20)	1543 (5.67)	8450 (17.36)
**Aspirin Prescription**	5344 (1.05)	4363 (1.00)	246 (0.90)	735 (1.51)
**Menopause status**^c^				
**Premenopausal**	128,510 (25.19)	113,942 (26.24)	10,681 (39.25)	3887 (7.99)
**Perimenopausal**	14,768 (2.90)	12,889 (2.97)	1022 (3.76)	857 (1.76)
**Postmenopausal**	157,587 (30.89)	130,529 (30.06)	7682 (28.23)	19,376 (39.81)
**History of diabetes**	30,007 (5.88)	25,356 (5.84)	1468 (5.39)	3183 (6.54)

BMI: body mass index; SD: standard deviation; MET: metabolic equivalent of task.

a 2.54% urban residents and 15.06% rural residents rarely or never brushed teeth, respectively.

b median (the 25th quartile, the 75th quartile).

c in women only.

### Oral health status and total cancer risk

**Figure 1:**
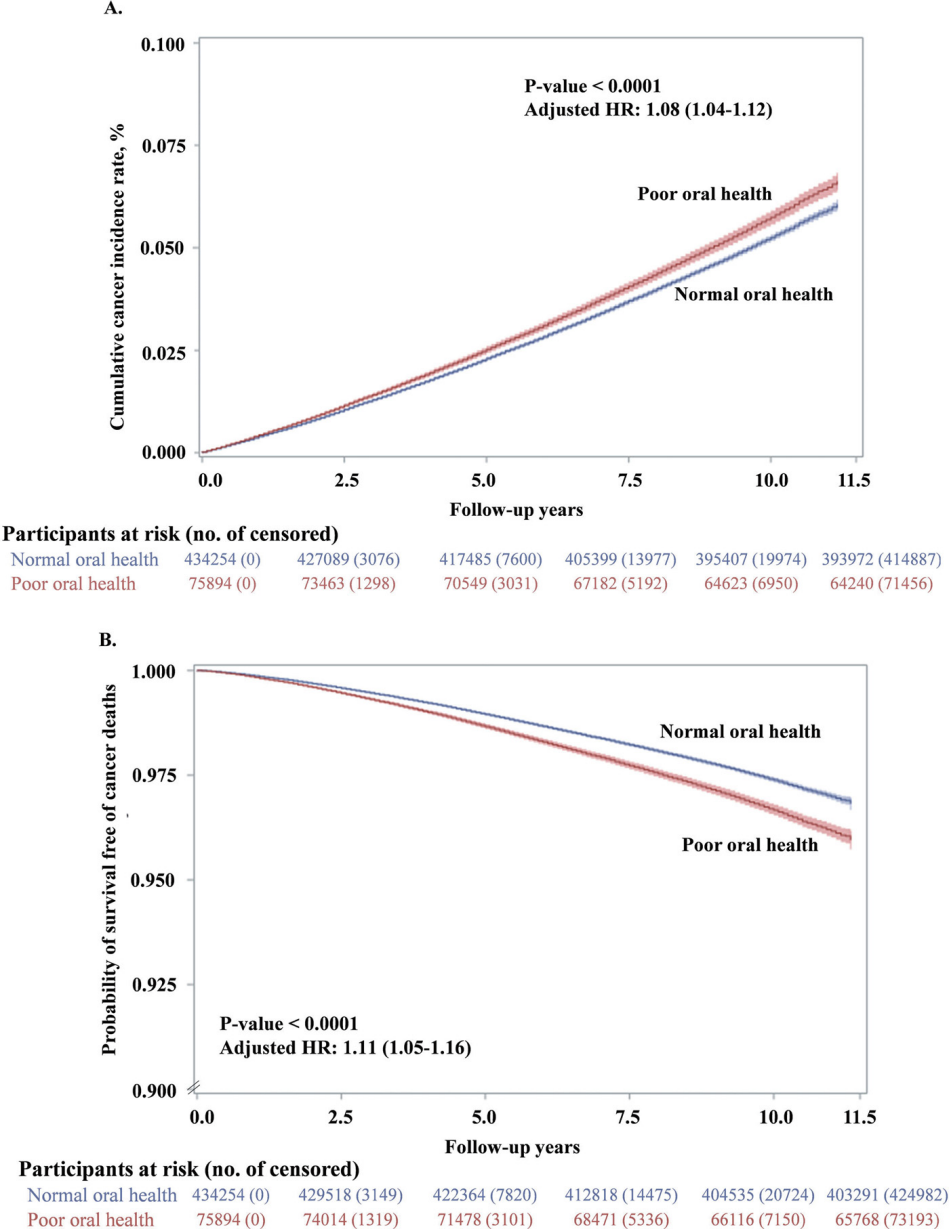
Age-adjusted cumulative incidence of total cancer and probability of survival free of cancer deaths by oral health status over 10 years. Cumulative probability of cancer incidence and survival for the participants who reported normal oral health (Blue) and poor oral health (Red). Hazard ratios for total cancer mortality compare participants with poor oral health to those with normal oral health. The 95% confidence intervals (CIs) are shown for each curve. Analyses were adjusted for age. (A) Cumulative probability of cancer incidence. (B) Probability of survival free of cancer deaths.

After a total of 4602,743 person-years (median of 9.17 years and range: 0.1∼11.5 years) of follow-up, we identified 23,805 new cancer cases and 11,973 cancer deaths, respectively. We observed significantly higher risks of both incident cancer (adjusted HR: 1.08, 95% CI: 1.04 to 1.12, Table 2 and Figure 1**A**) and cancer death (adjusted HR: 1.10, 95% CI: 1.05 to 1.16) in participants with poor oral health (Table 2 and Figure 1**B**). These results were consistent with the results from the crude models (**Supplemental Table S2**). Over 10% higher risks of cancer incidence (adjusted HR: 1.12, 95% CI: 1.07 to 1.17) and cancer mortality (adjusted HR: 1.11, 95% CI: 1.05 to 1.18) were observed in participants who rarely/never brushed teeth versus those with normal oral health. No significant association was observed between gum always bleeding and total cancer incidence or death (**Supplemental Table S2**). Sensitivity analyses by excluding cancer cases or deaths within the first 2 years and considering competing risks from deaths of other causes did not alter the results substantially (**Supplemental Table S2**).

**Table 2:** Associations between oral health status and risk of total and site-specific cancer in the CKB cohort.

Cancer site	Cases/ Incidence rate^a^	Cases^b^	Cancer Incidence, adjusted HR (95% CI)^c^		
			Poor oral health	Gum bleeding	Rarely or never brush teeth
**Total cancer**	23,805/5.23	19,367/958/3480	1.08 (1.04–1.12)	1.00 (0.94–1.07)	1.12 (1.07–1.17)
**Lung**	5007/1.09	4160/163/684	1.04 (0.95–1.13)	1.00 (0.86–1.18)	1.05 (0.95–1.15)
**Breast (females)**	2025/0.74	1781/131/113	0.97 (0.84–1.12)	0.96 (0.80–1.15)	0.98 (0.79–1.22)
**Stomach**	2964/0.64	2253/101/610	1.10 (1.00–1.22)	1.00 (0.82–1.23)	1.13 (1.01–1.27)
**Esophageal**	2119/0.46	1178/37/904	1.19 (1.07–1.33)	0.72 (0.52–1.00)	1.27 (1.13–1.43)
**Liver**	2565/0.56	2034/136/395	1.18 (1.06–1.32)	1.56 (1.31–1.86)	1.04 (0.91–1.19)
**Colorectal**	2678/0.58	2331/97/250	0.83 (0.73–0.93)	0.87 (0.71–1.07)	0.80 (0.69–0.93)
**Cervis uteri**	987/0.36	886/50/51	0.92 (0.74–1.15)	1.04 (0.78–1.40)	0.81 (0.59–1.11)
**Pancreas**	706/0.15	585/26/95	0.94 (0.76–1.17)	0.89 (0.60–1.33)	0.96 (0.75–1.24)
**Head & neck**	614/0.13	536/28/50	1.09 (0.84–1.41)	1.08 (0.73–1.58)	1.10 (0.79–1.54)
**Leukemia and lymphoma**	1239/0.27	1080/51/108	0.86 (0.71–1.03)	0.97 (0.73–1.29)	0.80 (0.63–1.00)
Cancer site	Deaths/ Mortality rate^a^	Deaths^b^	Cancer Mortality, adjusted HR (95% CI)^b^		
			Poor oral health	Gum bleeding	Rarely or never brush teeth
**Total cancer**	11,973/2.60	9272/419/2282	1.10 (1.05–1.16)	1.07 (0.97–1.18)	1.11 (1.05–1.18)
**Lung**	3213/0.70	2637/91/485	1.01 (0.91–1.11)	0.95 (0.77–1.18)	1.02 (0.91–1.14)
**Breast (females)**	262/0.10	217/17/28	1.22 (0.86–1.73)	1.18 (0.72–1.94)	1.25 (0.78–1.99)
**Stomach**	1594/0.35	1159/48/387	1.12 (0.98–1.27)	1.01 (0.75–1.35)	1.14 (0.99–1.32)
**Esophageal**	1238/0.27	679/26/533	1.29 (1.12–1.49)	0.98 (0.66–1.46)	1.34 (1.16–1.56)
**Liver**	1826/0.40	1413/89/324	1.20 (1.05–1.36)	1.50 (1.21–1.87)	1.10 (0.94–1.27)
**Colorectal**	855/0.19	715/28/112	0.90 (0.73–1.10)	0.87 (0.59–1.29)	0.91 (0.72–1.15)
**Cervis uteri**	158/0.06	134/11/13	1.23 (0.77–1.96)	1.62 (0.87–3.04)	0.97 (0.52–1.83)
**Pancreas**	500/0.11	409/17/74	0.94 (0.73–1.21)	0.80 (0.49–1.33)	0.99 (0.75–1.33)
**Head & neck**	171/0.04	140/7/24	1.17 (0.76–1.81)	1.12 (0.52–2.41)	1.19 (0.72–1.99)
**Leukemia and lymphoma**	400/0.09	347/12/41	0.78 (0.57–1.08)	0.70 (0.39–1.25)	0.82 (0.56–1.20)

a Incidence or mortality rate per 1000 person-years.

b The number of cases or deaths in normal oral health group/gum bleeding group/ rarely or never brushing teeth group.

c The reference category was “Normal oral health”. Cox regression model was adjusted for age (continuous), sex (male, female), body mass index (BMI, continuous), study sites (10 sites), education level (no formal school, primary or middle school, high school and above), marital status (married, other), household income per year (< ¥10,000, ¥10,000–19,999, ¥20,000–34,999, or ≥ ¥35,000), alcohol consumption (non-drinker, occasional drinker, former drinker, or regular drinker), smoking status (never smoker, occasional smoker, former smoker, or regular smoker), physical activity in metabolic equivalent tasks (MET) hours a day (continuous), aspirin prescription for CVD (no, yes, or missing), menopausal status (pre-menopausal or post-menopausal, women only), personal history of diabetes (no, yes), and family history of cancer (no, yes). The HRs for poor oral health, gum bleeding and rarely or never brush teeth were calculated according to independent models.

### Oral health status and site-specific cancer risk

Poor oral health was associated with higher incidence risks for stomach (adjusted HR: 1.10, 95% CI: 1.00 to 1.22), esophageal (adjusted HR: 1.19, 95% CI: 1.07 to 1.33), and liver (adjusted HR: 1.18, 95% CI: 1.06 to 1.32), respectively (Table 2). Cancer site-specific mortality for esophageal (adjusted HR: 1.29, 95% CI: 1.12–1.49), and liver (adjusted HR: 1.20, 95% CI: 1.05–1.36) was also elevated among those with poor oral health, respectively (Table 2). In contrast, poor oral health was associated with a lower HR of incident colorectal cancer (adjusted HR: 0.83; 95% CI: 0.73–0.93, Table 2). No statistically significant associations were observed for cancer risk of the lung, female breast, cervix uteri, pancreas, head and neck, or leukemia and lymphoma (Table 2). Compared with participants with normal oral health, those who rarely or never brushed teeth had higher risks of stomach and esophageal cancer, while those with gum bleeding had a higher risk of liver cancer (Table 2).

### Subgroup analyses

**Figure 2:**
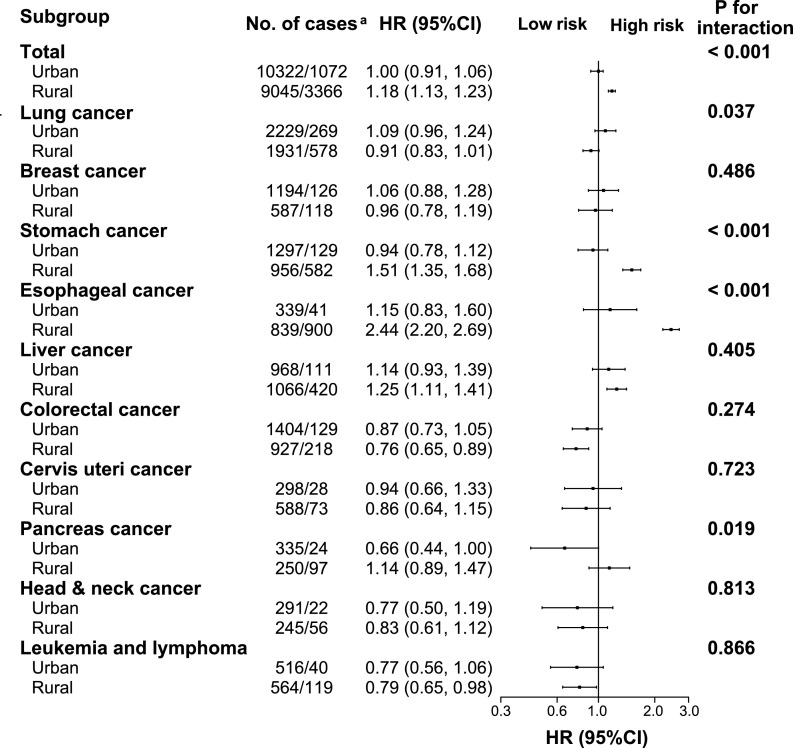
Subgroup analysis of associations between oral health status and site-specific cancer incidence according to residential area. Cancer incidence rate are crude rate (per 1000 person-years). Hazard ratios for total cancer mortality compare participants with poor oral health to those with normal oral health. Forest plot showing HRs (log scale) and 95% CI (horizontal line). Analyses were adjusted for adjusted for age, sex, body mass index, study sites, education level, marital status, household income per year, alcohol consumption, smoking status, physical activity in metabolic equivalent tasks (MET) hours a day, aspirin prescription for CVD, menopausal status, personal history of diabetes, and family history of cancer, as appropriate. ^a^ The number of cases in normal/poor oral health groups.

**Figure 3:**
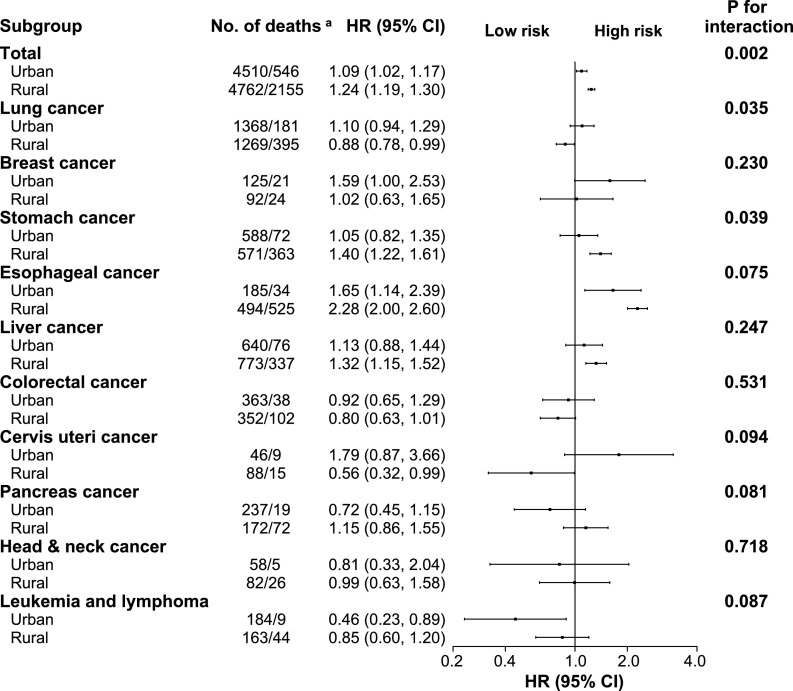
Subgroup analysis of associations between oral health status and site-specific cancer mortality according to residential area. Cancer mortality rate are crude rate (per 1000 person-years). Hazard ratios for total cancer mortality compare participants with poor oral health to those with normal oral health. Forest plot showing HRs (log scale) and 95% CI (horizontal line). Analyses were adjusted for adjusted for age, sex, body mass index, study sites, education level, marital status, household income per year, alcohol consumption, smoking status, physical activity in metabolic equivalent tasks (MET) hours a day, aspirin prescription for CVD, menopausal status, personal history of diabetes, and family history of cancer, as appropriate. ^a^ The number of deaths in normal/poor oral health groups.

**Figure 4:**
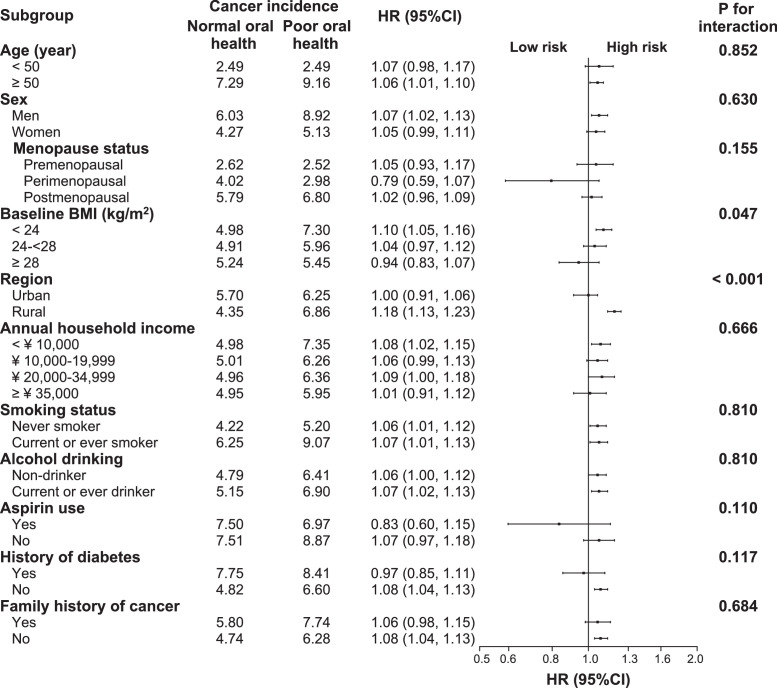
Subgroup analysis of associations between oral health status and total cancer incidence according to potential risk factors. Hazard ratios for total cancer incidence compare participants with poor oral health to those with normal oral health. Forest plot showing HRs (log scale) and 95% CI (horizontal line). Analyses were adjusted for adjusted for age, sex, body mass index, study sites, education level, marital status, household income per year, alcohol consumption, smoking status, physical activity in metabolic equivalent tasks (MET) hours a day, aspirin prescription for CVD, menopausal status, personal history of diabetes, and family history of cancer, as appropriate.

The association between poor oral health and risk of total cancer incidence was stronger in rural residents (adjusted HR: 1.18, 95% CI: 1.13–1.23) than in urban residents (adjusted HR: 1.00, 95% CI: 0.91–1.06, *P*-value for interaction < 0.001, Figure 2). This modifying effect of residential status was also seen for total cancer mortality (*P* = 0.002) (Figure 3). The positive associations between poor oral health and total incidence or mortality were generally similar across subgroups stratified according to age, sex, menopause status, annual household income, smoking status, alcohol consumption, aspirin use, history of diabetes, or family history of cancer (*P-*values for interaction all > 0.05, Figures. 4 **and** 5).

**Figure 5:**
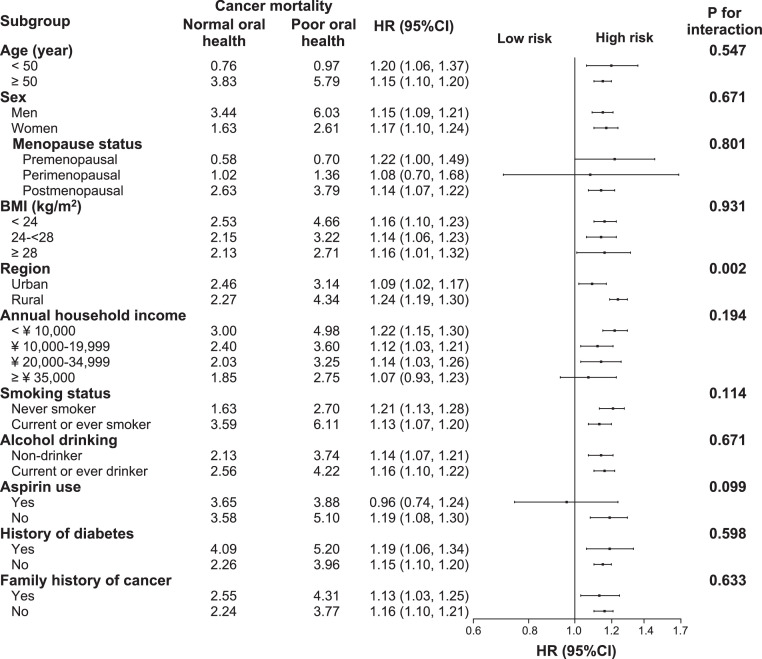
Subgroup analysis of associations between oral health status and total cancer mortality according to potential risk factors. Hazard ratios for total cancer mortality compare participants with poor oral health to those with normal oral health. Forest plot showing HRs (log scale) and 95% CI (horizontal line). Analyses were adjusted for adjusted for age, sex, body mass index, study sites, education level, marital status, household income per year, alcohol consumption, smoking status, physical activity in metabolic equivalent tasks (MET) hours a day, aspirin prescription for CVD, menopausal status, personal history of diabetes, and family history of cancer, as appropriate.

For esophageal and stomach cancer, the associations of poor oral health with risk of cancer incidence and mortality existed only in rural residents but not in urban residents (**Supplemental Tables S3 and S4**). For liver and colorectal cancer, no significant interaction factors were detected for cancer incidence or mortality (**Supplemental Tables S5 and S6**).

## Discussion

In this large-scale prospective cohort study, we found that poor oral health, compared to normal oral health, was significantly associated with higher risks of both cancer incidence and mortality. The association was stronger for cancers of the digestive system, such as esophageal, stomach and liver. Furthermore, we found that residential status modified the associations of poor oral health with cancer risk. The above findings were also robust according to various sensitivity analyses.

The positive association between poor oral health and total cancer risk observed here is consistent with findings from previous cohort studies, including studies conducted in Japan,^27^ U.S.,^12^^,^^16^^,^^17^^,^^28^^,^^29^ Sweden^30^ and China.^5^ Although different studies used different surrogate markers (e.g. periodontal disease, tooth number or tooth brushing) as indicators of poor oral health, all the above studies suggested a positive link between oral health status and overall cancer risk.

Our findings also agree with prior evidence showing different risks of poor oral health status on different site-specific cancers. In the U.S. Health Professionals Follow-Up Study, periodontal disease was associated with cancer risks of the pancreatic,^31^ lung,^12^ kidney,^12^ bladder,^29^ esophageal and oropharyngeal,^29^ and hematological cancers.^29^^,^^32^ However, only esophageal and stomach cancers were reported to be associated with periodontal disease (measured by tooth loss) in a high-risk Chinese population.^33^^,^^34^ Likewise, we also found higher risks of esophageal and stomach cancer in participants with poor oral health in the CKB cohort. Moreover, we found an elevated risk of liver cancer for participants with poor oral health. The association of poor oral health with liver cancer risk was weaker compared to esophageal cancer, but stronger than other cancers. Compared to the U.S., China has a heavy burden of liver, stomach and esophageal cancer, especially in rural areas.^35^^,^^36^ Differences observed in the cancer spectrum between various population groups are likely the result of a complex interplay of genetic, environmental, and social factors. These may also explain why the associations between poor oral health and cancer varied by region of residence.

Several potential mechanisms may explain the association between poor oral health and digestive system cancers. Different types of poor oral health may have different origins. For example, lack of oral hygiene can lead to periodontitis, although it can occur for different reasons and in the presence of good oral hygiene. Periodontal disease is directly and indirectly mediated by oral bacteria, which can contribute to tumorigenesis in the oral cavity as well as in distant body sites.^37^^,^^38^ The emerging oral-gut-liver axis suggests that the oral microbiota and inflammation connect oral, gastrointestinal and liver disease.^39^ *P. gingivalis*, the most important causative pathogen of periodontitis, was observed to be associated with higher risk of esophageal squamous cell carcinoma (ESCC)^40^ and worse ESCC prognosis.^41^ Besides, *P. gingivalis, T. forsythia, T. denticola*, and *A. actinomycetemcomitans* may contribute to the development of cancer or precancerous lesions of stomach, esophageal cancer and hepatocellular carcinoma.^42^, ^43^, ^44^, ^45^ These periodontal pathogens are able to inhibit oral epithelial innate immune responses through various mechanisms and to escape from host immune reaction, which may further lead to systemic inflammation.^46^ Moreover, periodontal therapy could increase the efficiency of H. pylori eradication and the non-recurrence rate of stomach H. pylori.^47^ Therefore, oral microbiota dysbiosis could be a possible mediator linked poor oral health to tumor initiation, promotion and progression. Another type of poor oral health is gum bleeding, which is a sign of gingivitis, or inflammation of the gums. Our results suggested that it was the periodontal disease/gum bleeding that drove the liver cancer association but was poor oral hygiene for stomach and esophagus cancer. Other mechanisms of oral health in cancer development have been postulated in the previous studies, including the potential toxic effects of nitrate-nitrite-nitrosamine reaction promoted by the oral flora,^33^ vitamin D deficiency in relation to periodontal disease and carcinogenesis,^48^ and oral bacteria-induced chronic inflammation that may lead to cancer.^38^ These hypotheses merit further investigation.

In this study, we found an inverse association between poor oral health and colorectal cancer incidence. The significant association was observed in participants who rarely or never brushing teeth rather than who have bleeding gums, and in rural residents rather than urban residents. One possible explanation is that certain oral microbiota type, such as *Lachnospiraceae*, was negatively associated with the colonization of colonic tissue with oral-like bacterial networks in colorectal cancer development.^49^ However, the specific mechanism of oral bacteria in colorectal cancer carcinogenesis is unknown. Additionally, other unexamined factors, such as nutritional status, could confound the result. The poorer residents who happens not to brush regularly might suffer from nutrient deficiency, which increases risks of esophageal and stomach cancer. However, they may tend to have less processed foods, thus protecting them from colon cancer. Although we have controlled many potential confounders and applied subgroup analyses, unknown confounders could exist. Alternatively, these findings might be a result of chance since we studied a large number of cancer sites. Thus, the association between poor oral health and colorectal cancer risk should be interpreted with caution.

The prospective design of the CKB study, including a geographically widespread Chinese population, and a large sample size provided us with sufficient statistical power to analyze individual types of cancer. To avoid potential reverse causality bias, we excluded participants who developed or died from cancer during the first two years of follow-up in sensitivity analyses. Moreover, we also considered competing risk for total cancer risk because a majority of the study participants (69%) died due to other causes than cancer. The consistent results from the sensitivity analyses indicate the robustness of our findings. However, several limitations should be noted. First, the observational design of the study limited causal inference of poor oral health in carcinogenesis. Second, the self-reported measures of gum bleeding and tooth brushing are not perfect indicators of oral health, and may introduce misclassification, though clinical dental measurements for such a large cohort would not be practical. Third, although we carefully controlled for established and potential risk factors for cancer, we were unable to obtain detailed information regarding dental visit history, tooth decay, drug abuse and brush teeth frequency, and control their possible confounding effects. Fourth, potential confounders such as HBV infection and cancer screening accessibility were not available. Fifth, selection bias and unobserved confounding factors are possible.

In conclusion, in this large nationwide cohort of over 0.5 million Chinese adults, we found that poor oral health was associated with a higher risk of total cancer, especially for cancers of the digestive system, such as esophageal, stomach and liver. Residential status also modified the associations. These findings suggest that promotion of oral health in the general population, especially for rural residents, could help reduce cancer risk. Further studies are warranted to elucidate the underlying mechanisms of the observed association.

## Contributors

X.Z. and H.D. designed the study, conducted the literature review and drafted the manuscript. H.D. accessed the data and was responsible for the raw data associated with the study. B.L. and H.S.L. provided methodological support and contributed to the interpretation of the data. H.S.L. and K.C. gave critical comments. All authors reviewed the article, read the final manuscript and approved the submission.

### Data sharing statement

China Kadoorie Biobank data underlying this article can be accessed at https://www.ckbiobank.org/site/Data+Access.

### Funding

Supported by grants (2021YFC2500400, 2016YFC0900500, 2016YFC0900501, 2016YFC0900504) from the National Key Research and Development Program of China, grants from the Kadoorie Charitable Foundation in Hong Kong and grants (088158/Z/09/Z, 104085/Z/14/Z, 202922/Z/16/Z) from Wellcome Trust in the UK. CKB is supported by the Kadoorie Charitable Foundation (KCF) in Hong Kong.

## Declaration of interests

The authors declare no conflicts of interest.

## Appendix Supplementary materials

Image, application 1

Image, application 1

## References

[1] Kassebaum N.J., Smith A.G.C., Bernabe E. (2017). Global, regional, and national prevalence, incidence, and disability-adjusted life years for oral conditions for 195 countries, 1990–2015: a systematic analysis for the global burden of diseases, injuries, and risk factors. J Dent Res.

[2] Peres M.A., Macpherson L.M.D., Weyant R.J. (2019). Oral diseases: a global public health challenge. Lancet.

[3] Jiao J., Jing W., Si Y. (2021). The prevalence and severity of periodontal disease in Mainland China: data from the fourth national oral health survey (2015-2016). J Clin Periodontol.

[4] Zhao Q., Wang S.B., Xu G. (2019). Periodontal health: a national cross-sectional study of knowledge, attitudes and practices for the public oral health strategy in China. J Clin Periodontol.

[5] Zhuang Z., Gao M., Lv J. (2021). Associations of toothbrushing behaviour with risks of vascular and nonvascular diseases in Chinese adults. Eur J Clin Investig.

[6] Munoz Aguilera E., Suvan J., Orlandi M. (2021). Association between periodontitis and blood pressure highlighted in systemically healthy individuals: results from a nested case-control study. Hypertension.

[7] LaMonte M.J., Genco R.J., Hovey K.M. (2017). History of periodontitis diagnosis and edentulism as predictors of cardiovascular disease, stroke, and mortality in postmenopausal women. J Am Heart Assoc.

[8] de Oliveira C., Watt R., Hamer M. (2010). Toothbrushing, inflammation, and risk of cardiovascular disease: results from Scottish health survey. BMJ.

[9] Su L., Liu W., Xie B. (2016). Toothbrushing, blood glucose and HbA1c: findings from a random survey in Chinese population. Sci Rep.

[10] Chen Y., Yang Y.C., Zhu B.L. (2020). Association between periodontal disease, tooth loss and liver diseases risk. J Clin Periodontol.

[11] Si Y., Fan H., Song Y. (2012). Association between periodontitis and chronic obstructive pulmonary disease in a Chinese population. J Periodontol.

[12] Michaud D.S., Liu Y., Meyer M. (2008). Periodontal disease, tooth loss, and cancer risk in male health professionals: a prospective cohort study. Lancet Oncol.

[13] Nwizu N., Wactawski-Wende J., Genco R.J. (2020). Periodontal disease and cancer: epidemiologic studies and possible mechanisms. Periodontology 2000.

[14] Gerlovin H., Michaud D.S., Cozier Y.C. (2019). Oral health in relation to pancreatic cancer risk in African American women. Cancer Epidemiol Biomark Prev.

[15] Heikkila P., But A., Sorsa T. (2018). Periodontitis and cancer mortality: register-based cohort study of 68,273 adults in 10-year follow-up. Int J Cancer.

[16] Nwizu N.N., Marshall J.R., Moysich K. (2017). Periodontal disease and incident cancer risk among postmenopausal women: results from the women's health initiative observational cohort. Cancer Epidemiol Biomark Prev.

[17] Michaud D.S., Lu J., Peacock-Villada A.Y. (2018). Periodontal disease assessed using clinical dental measurements and cancer risk in the ARIC study. J Natl Cancer Inst.

[18] Chen Z., Lee L., Chen J. (2005). Cohort profile: the kadoorie study of chronic disease in China (KSCDC). Int J Epidemiol.

[19] Chen Z., Chen J., Collins R. (2011). China kadoorie biobank of 0.5 million people: survey methods, baseline characteristics and long-term follow-up. Int J Epidemiol.

[20] Li L.M., Lv J., Guo Y. (2012). The China kadoorie biobank: related methodology and baseline characteristics of the participants. Zhonghua Liu Xing Bing Xue Za Zhi.

[21] Zhang Q., Li L., Smith M. (2013). Exhaled carbon monoxide and its associations with smoking, indoor household air pollution and chronic respiratory diseases among 512,000 Chinese adults. Int J Epidemiol.

[22] Im P.K., Millwood I.Y., Kartsonaki C. (2021). Alcohol drinking and risks of total and site-specific cancers in China: a 10-year prospective study of 0.5 million adults. Int J Cancer.

[23] Pang Y., Lv J., Kartsonaki C. (2020). Association of physical activity with risk of hepatobiliary diseases in China: a prospective cohort study of 0.5 million people. Br J Sports Med.

[24] Pan R., Zhu M., Yu C. (2017). Cancer incidence and mortality: a cohort study in China, 2008–2013. Int J Cancer.

[25] Kleinbaum D.G., Klein M. (2012).

[26] Fine J.P., Gray R.J. (1999). A proportional hazards model for the subdistribution of a competing risk. J Am Stat Assoc.

[27] Goto Y., Wada K., Uji T. (2020). Number of teeth and all-cause and cancer mortality in a Japanese community: the Takayama study. J Epidemiol.

[28] Hujoel P.P., Drangsholt M., Spiekerman C. (2003). An exploration of the periodontitis-cancer association. Ann Epidemiol.

[29] Michaud D.S., Kelsey K.T., Papathanasiou E. (2016). Periodontal disease and risk of all cancers among male never smokers: an updated analysis of the health professionals follow-up study. Ann Oncol Off J Eur Soc Med Oncol.

[30] Arora M., Weuve J., Fall K. (2010). An exploration of shared genetic risk factors between periodontal disease and cancers: a prospective co-twin study. Am J Epidemiol.

[31] Michaud D.S., Joshipura K., Giovannucci E. (2007). A prospective study of periodontal disease and pancreatic cancer in US male health professionals. J Natl Cancer Inst.

[32] Bertrand K.A., Shingala J., Evens A. (2017). Periodontal disease and risk of non-Hodgkin lymphoma in the health professionals follow-up study. Int J Cancer.

[33] Abnet C.C., Qiao Y.L., Dawsey S.M. (2005). Tooth loss is associated with increased risk of total death and death from upper gastrointestinal cancer, heart disease, and stroke in a Chinese population-based cohort. Int J Epidemiol.

[34] Abnet C.C., Qiao Y.L., Mark S.D. (2001). Prospective study of tooth loss and incident esophageal and gastric cancers in China. Cancer Causes Control CCC.

[35] Qiu H., Cao S., Xu R. (2021). Cancer incidence, mortality, and burden in China: a time-trend analysis and comparison with the United States and United Kingdom based on the global epidemiological data released in 2020. Cancer Commun (Lond).

[36] Chen W., Zheng R., Baade P.D. (2016). Cancer statistics in China, 2015. CA Cancer J Clin.

[37] Hajishengallis G., Chavakis T. (2021). Local and systemic mechanisms linking periodontal disease and inflammatory comorbidities. Nat Rev Immunol.

[38] Teles F.R.F., Alawi F., Castilho R.M. (2020). Association or causation? Exploring the oral microbiome and cancer links. J Dent Res.

[39] Acharya C., Sahingur S.E., Bajaj J.S. (2017). Microbiota, cirrhosis, and the emerging oral-gut-liver axis. JCI Insight.

[40] Peters B.A., Wu J., Pei Z. (2017). Oral microbiome composition reflects prospective risk for esophageal cancers. Cancer Res.

[41] Gao S., Liu Y., Duan X. (2021). Porphyromonas gingivalis infection exacerbates oesophageal cancer and promotes resistance to neoadjuvant chemotherapy. Br J Cancer.

[42] Kawasaki M., Ikeda Y., Ikeda E. (2021). Oral infectious bacteria in dental plaque and saliva as risk factors in patients with esophageal cancer. Cancer.

[43] Zhang Z., Liu D., Liu S. (2020). The role of porphyromonas gingivalis outer membrane vesicles in periodontal disease and related systemic diseases. Front Cell Infect Microbiol.

[44] Sasaki N., Katagiri S., Komazaki R. (2018). Endotoxemia by porphyromonas gingivalis injection aggravates non-alcoholic fatty liver disease, disrupts glucose/lipid metabolism, and alters gut microbiota in mice. Front Microbiol.

[45] Sun J., Zhou M., Salazar C.R. (2017). Chronic periodontal disease, periodontal pathogen colonization, and increased risk of precancerous gastric lesions. J Periodontol.

[46] Groeger S., Meyle J. (2019). Oral mucosal epithelial cells. Front Immunol.

[47] Ren Q., Yan X., Zhou Y., Li W.X (2016). Periodontal therapy as adjunctive treatment for gastric helicobacter pylori infection. Cochrane Database Syst Rev.

[48] Grant W.B. (2008). Vitamin D, periodontal disease, tooth loss, and cancer risk. Lancet Oncol.

[49] Flemer B., Warren R.D., Barrett M.P. (2018). The oral microbiota in colorectal can cer is distinctive and predictive. Gut.

