# Supplementary material for: Poor oral health and risks of total and site-specific cancers in China: A prospective cohort study of 0.5 million adults
Source: eClinicalMedicine. 2022 Mar 5;45:101330. doi: 10.1016/j.eclinm.2022.101330 (PMC8902628; doi:10.1016/j.eclinm.2022.101330)
Supplement: Supplementary file 1 [file mmc1.docx]

Caption for supplementary material

Supplementary Tables S1-S6
