# Supplementary material for: Poor oral health and risks of total and site-specific cancers in China: A prospective cohort study of 0.5 million adults
Source: eClinicalMedicine. 2022 Mar 5;45:101330. doi: 10.1016/j.eclinm.2022.101330 (PMC8902628; doi:10.1016/j.eclinm.2022.101330)
Supplement: Supplementary file 2 [file mmc2.docx]

# Supplementary Table S1. The agreement rate between the two surveys

| **Subgroups** | **Normal** | **Gum bleeding** | **Rarely or**  **never**  **brush teeth** | **Total** | **Kappa (95% CI)** |
| --- | --- | --- | --- | --- | --- |
| **Overall** |  |  |  |  | 0.598 (0.536-0.659) |
| **Normal** | 1041 | 42 | 41 | 1124 |  |
| **Gum bleeding** | 28 | 18 | 1 | 47 |  |
| **Rarely or never brush teeth** | 40 | 5 | 99 | 144 |  |
| **Total** | 1109 | 65 | 141 | 1315 |  |
| **Rural region** |  |  |  |  | 0.617 (0.547-0.687) |
| **Normal** | 462 | 9 | 38 | 509 |  |
| **Gum bleeding** | 8 | 5 | 1 | 14 |  |
| **Rarely or never brush teeth** | 39 | 4 | 99 | 142 |  |
| **Total** | 509 | 18 | 138 | 665 |  |
| **Urban region** |  |  |  |  | 0.274 (0.144-0.405) |
| **Normal** | 579 | 33 | 3 | 615 |  |
| **Gum bleeding** | 20 | 13 | 0 | 33 |  |
| **Rarely or never brush teeth** | 1 | 1 | 0 | 2 |  |
| **Total** | 600 | 47 | 3 | 650 |  |

# Supplementary Table S2. Association of oral health status with risk of total cancer incidence and mortality

| Outcome | No. of events | Incidence/  Mortality rate ^a^ | Crude HR (95% CI) | *P* | Adjusted HR (95% CI) ^b^ | *P* |
| --- | --- | --- | --- | --- | --- | --- |
| Cancer incidence |  |  |  |  |  |  |
| Normal oral health | 19367 | 4.98 | Ref. |  | Ref. |  |
| Poor oral health | 4438 | 6.70 | 1.35 (1.31-1.40) | < 0.001 | 1.08 (1.04-1.12) | < 0.001 |
| Gum bleeding | 958 | 3.84 | 0.77 (0.72-0.82) | < 0.001 | 1.00 (0.94-1.07) | 0.885 |
| Brush teeth rarely or never | 3480 | 8.44 | 1.71 (1.64-1.77) | < 0.001 | 1.12 (1.07-1.17) | < 0.001 |
| Cancer death |  |  |  |  |  |  |
| Normal oral health | 9272 | 2.36 | Ref. |  | Ref. |  |
| Poor oral health | 2701 | 4.03 | 1.73 (1.65-1.80) | < 0.001 | 1.11 (1.05-1.16) | < 0.001 |
| Gum bleeding | 419 | 1.66 | 0.71 (0.65-0.79) | < 0.001 | 1.07 (0.97-1.18) | 0.18 |
| Brush teeth rarely or never | 2282 | 5.45 | 2.34 (2.24-2.45) | < 0.001 | 1.11 (1.05-1.18) | < 0.001 |
| Cancer incidence (Sensitivity analysis ^c^) |  |  |  |  |  |  |
| Normal oral health | 16068 | 4.14 | Ref. |  | Ref. |  |
| Poor oral health | 3499 | 5.30 | 1.30 (1.25-1.35) | < 0.001 | 1.08 (1.04-1.13) | < 0.001 |
| Gum bleeding | 793 | 3.18 | 0.76 (0.72-0.83) | < 0.001 | 1.01 (0.94-1.08) | 0.825 |
| Brush teeth rarely or never | 2706 | 6.59 | 1.62 (1.55-1.68) | < 0.001 | 1.12 (1.07-1.17) | < 0.001 |
| Cancer death (Sensitivity analysis ^c^) |  |  |  |  |  |  |
| Normal oral health | 8027 | 2.04 | Ref. |  | Ref. |  |
| Poor oral health | 2270 | 3.39 | 1.68 (1.61-1.77) | < 0.001 | 1.15 (1.09-1.21) | < 0.001 |
| Gum bleeding | 365 | 1.45 | 0.72 (0.65-0.80) | < 0.001 | 1.11 (1.00-1.24) | 0.05 |
| Brush teeth rarely or never | 1905 | 4.57 | 2.27 (2.16-2.39) | < 0.001 | 1.16 (1.09-1.23) | < 0.001 |
| Cancer incidence (Sensitivity analysis ^d^) |  |  |  |  |  |  |
| Normal oral health | 19367 | 4.98 | Ref. |  | Ref. |  |
| Poor oral health | 4438 | 6.70 | 1.39 (1.35-1.44) | < 0.001 | 1.11 (1.07-1.15) | < 0.001 |
| Gum bleeding | 958 | 3.84 | 0.77 (0.72-0.82) | < 0.001 | 1.01 (0.94-1.07) | 0.873 |
| Brush teeth rarely or never | 3480 | 8.44 | 1.80 (1.73-1.86) | < 0.001 | 1.15 (1.10-1.21) | < 0.001 |
| Cancer death (Sensitivity analysis ^d^) |  |  |  |  |  |  |
| Normal oral health | 9272 | 2.36 | Ref. |  | Ref. |  |
| Poor oral health | 2701 | 4.03 | 1.68 (1.61-1.75) | < 0.001 | 1.06 (1.01-1.12) | 0.015 |
| Gum bleeding | 419 | 1.66 | 0.72 (0.65-0.79) | < 0.001 | 1.06 (0.96-1.18) | 0.153 |
| Brush teeth rarely or never | 2282 | 5.45 | 2.23 (2.13-2.33) | < 0.001 | 1.06 (1.01-1.13) | 0.001 |

^a^ Incidence/Mortality rate per 1,000 person-years.

^b^ Adjusted for age (continuous), sex (male, female), body mass index (BMI, continuous), study sites (10 sites), education level (no formal school, primary or middle school, high school and above), marital status (married, other), household income per year (< ¥10,000, ¥10,000–19,999, ¥20,000–34,999, or ≥ ¥35,000), alcohol consumption (non-drinker, occasional drinker, former drinker, or regular drinker), smoking status (never smoker, occasional smoker, former smoker, or regular smoker), physical activity in metabolic equivalent tasks (MET) hours a day (continuous), aspirin prescription for CVD (no, yes, or missing), menopausal status (pre-menopausal or post-menopausal, women only), personal history of diabetes (no, yes), and family history of cancer (no, yes).

^c^ Sensitivity analysis by excluding participants who developed cancer or died from cancer during the first 2 years of follow-up.

^d^ Sensitivity analysis by conducting competing risk regression analyses.

# Supplementary Table S3. Subgroup analysis of associations between oral health status and stomach cancer incidence and mortality

| **Subgroup** | **Incidence rate** | | | | **Mortality** | | | |
| --- | --- | --- | --- | --- | --- | --- | --- | --- |
|  | **HR^a^** | **LL** | **UL** | **P for  interaction** | **HR^a^** | **LL** | **UL** | **P for interaction** |
| **Age (year)** |  |  |  | 0.336 |  |  |  | 0.945 |
| **< 50** | 0.96 | 0.70 | 1.31 |  | 1.13 | 0.70 | 1.80 |  |
| **≥ 50** | 1.12 | 1.01 | 1.24 |  | 1.11 | 0.97 | 1.28 |  |
| **Sex** |  |  |  | 0.933 |  |  |  | 0.469 |
| **Men** | 1.10 | 0.97 | 1.24 |  | 1.07 | 0.92 | 1.25 |  |
| **Women** | 1.09 | 0.92 | 1.30 |  | 1.19 | 0.94 | 1.50 |  |
| **Menopause status** |  |  |  | 0.195 |  |  |  | 0.266 |
| **Premenopausal** | 0.76 | 0.43 | 1.33 |  | 0.58 | 0.21 | 1.62 |  |
| **Perimenopausal** | 0.55 | 0.16 | 1.87 |  | 1.51 | 0.36 | 6.42 |  |
| **Postmenopausal** | 1.12 | 0.93 | 1.36 |  | 1.21 | 0.94 | 1.55 |  |
| **Baseline BMI (kg/m^2^)** |  |  |  | 0.530 |  |  |  | 0.771 |
| **< 24** | 1.10 | 0.98 | 1.25 |  | 1.11 | 0.95 | 1.30 |  |
| **24-<28** | 1.17 | 0.97 | 1.41 |  | 1.18 | 0.91 | 1.53 |  |
| **≥ 28** | 0.93 | 0.64 | 1.35 |  | 0.96 | 0.58 | 1.61 |  |
| **Study site** |  |  |  | < 0.0001 |  |  |  | 0.037 |
| **Urban** | 0.93 | 0.78 | 1.12 |  | 1.05 | 0.82 | 1.35 |  |
| **Rural** | 1.51 | 1.34 | 1.68 |  | 1.40 | 1.22 | 1.61 |  |
| **Educational levels** |  |  |  | 0.402 |  |  |  | 0.694 |
| **No formal school** | 1.00 | 0.84 | 1.19 |  | 1.06 | 0.85 | 1.31 |  |
| **Primary or middle school** | 1.16 | 0.98 | 1.36 |  | 1.09 | 0.88 | 1.36 |  |
| **High school and above** | 1.15 | 0.95 | 1.39 |  | 1.23 | 0.95 | 1.60 |  |
| **Marital status** |  |  |  | 0.029 |  |  |  | 0.003 |
| **Currently married** | 1.14 | 1.03 | 1.27 |  | 1.21 | 1.05 | 1.40 |  |
| **Other** | 0.86 | 0.67 | 1.11 |  | 0.76 | 0.56 | 1.03 |  |
| **Household income, ¥ per year** |  |  |  | 0.889 |  |  |  | 0.625 |
| **< ¥10,000** | 1.10 | 0.95 | 1.28 |  | 1.18 | 0.99 | 1.41 |  |
| **¥10,000–19,999** | 1.11 | 0.92 | 1.35 |  | 1.05 | 0.80 | 1.37 |  |
| **¥20,000–34,999** | 1.17 | 0.92 | 1.50 |  | 1.20 | 0.83 | 1.73 |  |
| **≥ ¥35,000** | 1.00 | 0.74 | 1.36 |  | 0.88 | 0.54 | 1.44 |  |
| **Physical activity (MET hr/day)** |  |  |  | 0.116 |  |  |  | 0.413 |
| **< 12.29** | 1.11 | 0.95 | 1.29 |  | 1.16 | 0.95 | 1.42 |  |
| **12.29-< 25.3** | 1.33 | 1.12 | 1.57 |  | 1.18 | 0.93 | 1.49 |  |
| **≥ 25.3** | 1.02 | 0.84 | 1.23 |  | 0.96 | 0.74 | 1.24 |  |
| **Smoking status** |  |  |  | 0.667 |  |  |  | 0.653 |
| **Never smoker** | 1.13 | 0.96 | 1.32 |  | 1.16 | 0.93 | 1.43 |  |
| **Current or ever smoker** | 1.08 | 0.95 | 1.23 |  | 1.09 | 0.93 | 1.28 |  |
| **Alcohol drinking** |  |  |  | 0.439 |  |  |  | 0.641 |
| **Non-drinker** | 1.15 | 0.98 | 1.34 |  | 1.07 | 0.87 | 1.31 |  |
| **Current or ever drinker** | 1.06 | 0.93 | 1.21 |  | 1.14 | 0.96 | 1.35 |  |
| **Aspirin use** |  |  |  | 0.803 |  |  |  | 0.754 |
| **Yes** | 1.26 | 0.62 | 2.56 |  | 1.61 | 0.58 | 4.46 |  |
| **No** | 1.13 | 0.85 | 1.50 |  | 1.29 | 0.90 | 1.87 |  |
| **History of diabetes** |  |  |  | 1.000 |  |  |  | 0.681 |
| **Yes** | 1.10 | 0.99 | 1.22 |  | 1.24 | 0.75 | 2.05 |  |
| **No** | 1.10 | 0.73 | 1.64 |  | 1.10 | 0.96 | 1.26 |  |
| **Family history of cancer** |  |  |  | 0.448 |  |  |  | 0.614 |
| **Yes** | 1.04 | 0.84 | 1.28 |  | 1.20 | 0.90 | 1.60 |  |
| **No** | 1.14 | 1.01 | 1.28 |  | 1.10 | 0.94 | 1.28 |  |

**^a^** Adjusted for age (continuous), sex (male, female), body mass index (BMI, continuous), study sites (10 sites), education level (no formal school, primary or middle school, high school and above), marital status (married, other), household income per year (< ¥10,000, ¥10,000–19,999, ¥20,000–34,999, or ≥ ¥35,000), alcohol consumption (non-drinker, occasional drinker, former drinker, or regular drinker), smoking status (never smoker, occasional smoker, former smoker, or regular smoker), physical activity in metabolic equivalent tasks (MET) hours a day (continuous), aspirin prescription for CVD (no, yes, or missing), menopausal status (pre-menopausal or post-menopausal, women only), personal history of diabetes (no, yes), and family history of cancer (no, yes).

# Supplementary Table S4. Subgroup analysis of associations between oral health status and esophageal cancer incidence and mortality

| **Subgroup** | **Incidence rate** | | | | **Mortality** | | | |
| --- | --- | --- | --- | --- | --- | --- | --- | --- |
|  | **HR^a^** | **LL** | **UL** | **P for  interaction** | **HR^a^** | **LL** | **UL** | **P for interaction** |
| **Age (year)** |  |  |  | 0.195 |  |  |  | 0.825 |
| **< 50** | 0.96 | 0.68 | 1.35 |  | 1.35 | 0.83 | 2.20 |  |
| **≥ 50** | 1.20 | 1.07 | 1.35 |  | 1.27 | 1.09 | 1.47 |  |
| **Sex** |  |  |  | 0.371 |  |  |  | 0.732 |
| **Men** | 1.23 | 1.08 | 1.41 |  | 1.30 | 1.10 | 1.54 |  |
| **Women** | 1.11 | 0.92 | 1.33 |  | 1.23 | 0.94 | 1.61 |  |
| **Menopause status** |  |  |  | 0.240 |  |  |  | 0.646 |
| **Premenopausal** | 0.82 | 0.41 | 1.64 |  | 0.69 | 0.19 | 2.50 |  |
| **Perimenopausal** | 0.58 | 0.20 | 1.65 |  | 1.69 | 0.32 | 8.82 |  |
| **Postmenopausal** | 1.15 | 0.94 | 1.40 |  | 1.25 | 0.94 | 1.66 |  |
| **Baseline BMI (kg/m^2^)** |  |  |  | 0.961 |  |  |  | 0.783 |
| **< 24** | 1.18 | 1.03 | 1.35 |  | 1.25 | 1.05 | 1.48 |  |
| **24-<28** | 1.21 | 0.98 | 1.48 |  | 1.41 | 1.06 | 1.88 |  |
| **≥ 28** | 1.24 | 0.85 | 1.81 |  | 1.36 | 0.77 | 2.40 |  |
| **Study site** |  |  |  | < 0.0001 |  |  |  | 0.075 |
| **Urban** | 1.15 | 0.83 | 1.60 |  | 1.65 | 1.14 | 2.39 |  |
| **Rural** | 2.43 | 2.20 | 2.69 |  | 2.28 | 2.00 | 2.60 |  |
| **Educational levels** |  |  |  | 0.791 |  |  |  | 0.680 |
| **No formal school** | 1.24 | 1.00 | 1.54 |  | 1.17 | 0.89 | 1.52 |  |
| **Primary or middle school** | 1.14 | 0.98 | 1.34 |  | 1.36 | 1.10 | 1.68 |  |
| **High school and above** | 1.22 | 0.99 | 1.51 |  | 1.30 | 0.97 | 1.76 |  |
| **Marital status** |  |  |  | 0.674 |  |  |  | 0.726 |
| **Currently married** | 1.20 | 1.07 | 1.35 |  | 1.31 | 1.12 | 1.53 |  |
| **Other** | 1.12 | 0.83 | 1.52 |  | 1.22 | 0.84 | 1.76 |  |
| **Household income, ¥ per year** |  |  |  | 0.526 |  |  |  | 0.409 |
| **< ¥10,000** | 1.20 | 1.03 | 1.39 |  | 1.18 | 0.97 | 1.42 |  |
| **¥10,000–19,999** | 1.19 | 0.98 | 1.44 |  | 1.57 | 1.20 | 2.06 |  |
| **¥20,000–34,999** | 1.42 | 1.03 | 1.94 |  | 1.48 | 0.97 | 2.25 |  |
| **≥ ¥35,000** | 0.92 | 0.56 | 1.51 |  | 1.24 | 0.64 | 2.40 |  |
| **Physical activity (MET hr/day)** |  |  |  | 0.693 |  |  |  | 0.777 |
| **< 12.29** | 1.22 | 1.04 | 1.44 |  | 1.23 | 0.99 | 1.53 |  |
| **12.29-< 25.3** | 1.22 | 1.01 | 1.49 |  | 1.39 | 1.09 | 1.79 |  |
| **≥ 25.3** | 1.09 | 0.87 | 1.38 |  | 1.28 | 0.95 | 1.72 |  |
| **Smoking status** |  |  |  | 0.655 |  |  |  | 0.922 |
| **Never smoker** | 1.16 | 0.98 | 1.39 |  | 1.30 | 1.01 | 1.67 |  |
| **Current or ever smoker** | 1.22 | 1.06 | 1.39 |  | 1.28 | 1.08 | 1.53 |  |
| **Alcohol drinking** |  |  |  | 0.635 |  |  |  | 0.782 |
| **Non-drinker** | 1.11 | 0.89 | 1.39 |  | 1.22 | 0.90 | 1.64 |  |
| **Current or ever drinker** | 1.18 | 1.05 | 1.34 |  | 1.28 | 1.09 | 1.51 |  |
| **Aspirin use** |  |  |  | 0.195 |  |  |  | 0.551 |
| **Yes** | 0.73 | 0.31 | 1.71 |  | 0.76 | 0.25 | 2.36 |  |
| **No** | 1.28 | 0.91 | 1.81 |  | 1.12 | 0.71 | 1.78 |  |
| **History of diabetes** |  |  |  | 0.515 |  |  |  | 0.475 |
| **Yes** | 1.42 | 0.90 | 2.26 |  | 1.66 | 0.91 | 3.02 |  |
| **No** | 1.19 | 1.06 | 1.32 |  | 1.27 | 1.10 | 1.47 |  |
| **Family history of cancer** |  |  |  | 0.891 |  |  |  | 0.730 |
| **Yes** | 1.18 | 0.97 | 1.44 |  | 1.37 | 1.03 | 1.82 |  |
| **No** | 1.20 | 1.04 | 1.37 |  | 1.29 | 1.08 | 1.53 |  |

**^a^** Adjusted for age (continuous), sex (male, female), body mass index (BMI, continuous), study sites (10 sites), education level (no formal school, primary or middle school, high school and above), marital status (married, other), household income per year (< ¥10,000, ¥10,000–19,999, ¥20,000–34,999, or ≥ ¥35,000), alcohol consumption (non-drinker, occasional drinker, former drinker, or regular drinker), smoking status (never smoker, occasional smoker, former smoker, or regular smoker), physical activity in metabolic equivalent tasks (MET) hours a day (continuous), aspirin prescription for CVD (no, yes, or missing), menopausal status (pre-menopausal or post-menopausal, women only), personal history of diabetes (no, yes), and family history of cancer (no, yes).

# Supplementary Table S5. Subgroup analysis of associations between oral health status and liver cancer incidence and mortality

| **Subgroup** | **Incidence rate** | | | | **Mortality** | | | |
| --- | --- | --- | --- | --- | --- | --- | --- | --- |
|  | **HR^a^** | **LL** | **UL** | **P for  interaction** | **HR^a^** | **LL** | **UL** | **P for interaction** |
| **Age (year)** |  |  |  | 0.134 |  |  |  | 0.857 |
| **< 50** | 1.45 | 1.13 | 1.86 |  | 1.26 | 0.92 | 1.72 |  |
| **≥ 50** | 1.15 | 1.02 | 1.31 |  | 1.22 | 1.06 | 1.41 |  |
| **Sex** |  |  |  | 0.370 |  |  |  | 0.187 |
| **Men** | 1.14 | 0.99 | 1.31 |  | 1.12 | 0.96 | 1.32 |  |
| **Women** | 1.27 | 1.06 | 1.53 |  | 1.35 | 1.09 | 1.67 |  |
| **Menopause status** |  |  |  | 0.476 |  |  |  | 0.746 |
| **Premenopausal** | 1.53 | 0.97 | 2.39 |  | 1.14 | 0.77 | 2.59 |  |
| **Perimenopausal** | 0.70 | 0.20 | 2.47 |  | 0.93 | 0.25 | 3.48 |  |
| **Postmenopausal** | 1.27 | 1.04 | 1.56 |  | 1.41 | 1.12 | 1.78 |  |
| **Baseline BMI (kg/m^2^)** |  |  |  | 0.426 |  |  |  | 0.718 |
| **< 24** | 1.23 | 1.07 | 1.41 |  | 1.24 | 1.06 | 1.45 |  |
| **24-<28** | 1.13 | 0.91 | 1.40 |  | 1.10 | 0.86 | 1.41 |  |
| **≥ 28** | 0.94 | 0.61 | 1.46 |  | 1.20 | 0.73 | 1.98 |  |
| **Study site** |  |  |  | 0.432 |  |  |  | 0.267 |
| **Urban** | 1.14 | 0.93 | 1.39 |  | 1.13 | 0.88 | 1.44 |  |
| **Rural** | 1.25 | 1.11 | 1.41 |  | 1.32 | 1.15 | 1.52 |  |
| **Educational levels** |  |  |  | 0.722 |  |  |  | 0.067 |
| **No formal school** | 1.25 | 1.02 | 1.53 |  | 1.40 | 1.12 | 1.76 |  |
| **Primary or middle school** | 1.24 | 1.03 | 1.49 |  | 1.28 | 1.04 | 1.58 |  |
| **High school and above** | 1.13 | 0.93 | 1.37 |  | 0.97 | 0.76 | 1.23 |  |
| **Marital status** |  |  |  | 0.343 |  |  |  | 0.966 |
| **Currently married** | 1.21 | 1.07 | 1.36 |  | 1.20 | 1.04 | 1.38 |  |
| **Other** | 1.04 | 0.77 | 1.41 |  | 1.21 | 0.86 | 1.71 |  |
| **Household income, ¥ per year** |  |  |  | 0.923 |  |  |  | 0.595 |
| **< ¥10,000** | 1.25 | 1.06 | 1.47 |  | 1.27 | 1.06 | 1.53 |  |
| **¥10,000–19,999** | 1.14 | 0.92 | 1.41 |  | 1.06 | 0.82 | 1.36 |  |
| **¥20,000–34,999** | 1.21 | 0.92 | 1.59 |  | 1.36 | 0.99 | 1.87 |  |
| **≥ ¥35,000** | 1.17 | 0.84 | 1.64 |  | 1.24 | 0.83 | 1.87 |  |
| **Physical activity (MET hr/day)** |  |  |  | 0.555 |  |  |  | 0.659 |
| **< 12.29** | 1.20 | 1.02 | 1.43 |  | 1.20 | 0.99 | 1.46 |  |
| **12.29-< 25.3** | 1.07 | 0.87 | 1.32 |  | 1.09 | 0.85 | 1.38 |  |
| **≥ 25.3** | 1.25 | 1.01 | 1.55 |  | 1.28 | 1.00 | 1.65 |  |
| **Smoking status** |  |  |  | 0.385 |  |  |  | 0.475 |
| **Never smoker** | 1.26 | 1.06 | 1.49 |  | 1.28 | 1.04 | 1.57 |  |
| **Current or ever smoker** | 1.14 | 0.99 | 1.32 |  | 1.16 | 0.98 | 1.37 |  |
| **Alcohol drinking** |  |  |  | 0.772 |  |  |  | 0.621 |
| **Non-drinker** | 1.20 | 1.01 | 1.43 |  | 1.24 | 1.01 | 1.51 |  |
| **Current or ever drinker** | 1.16 | 1.00 | 1.34 |  | 1.16 | 0.98 | 1.37 |  |
| **Aspirin use** |  |  |  | 0.989 |  |  |  | 0.883 |
| **Yes** | 1.32 | 0.53 | 3.29 |  | 1.40 | 0.52 | 3.81 |  |
| **No** | 1.33 | 0.98 | 1.81 |  | 1.53 | 1.09 | 2.16 |  |
| **History of diabetes** |  |  |  | 0.500 |  |  |  | 0.215 |
| **Yes** | 1.33 | 0.94 | 1.89 |  | 1.55 | 1.06 | 2.25 |  |
| **No** | 1.16 | 1.04 | 1.31 |  | 1.16 | 1.01 | 1.33 |  |
| **Family history of diabetes** |  |  |  | 0.776 |  |  |  | 0.356 |
| **Yes** | 1.22 | 0.95 | 1.57 |  | 1.37 | 1.02 | 1.84 |  |
| **No** | 1.17 | 1.03 | 1.33 |  | 1.16 | 1.00 | 1.35 |  |

**^a^** Adjusted for age (continuous), sex (male, female), body mass index (BMI, continuous), study sites (10 sites), education level (no formal school, primary or middle school, high school and above), marital status (married, other), household income per year (< ¥10,000, ¥10,000–19,999, ¥20,000–34,999, or ≥ ¥35,000), alcohol consumption (non-drinker, occasional drinker, former drinker, or regular drinker), smoking status (never smoker, occasional smoker, former smoker, or regular smoker), physical activity in metabolic equivalent tasks (MET) hours a day (continuous), aspirin prescription for CVD (no, yes, or missing), menopausal status (pre-menopausal or post-menopausal, women only), personal history of diabetes (no, yes), and family history of cancer (no, yes).

# Supplementary Table S6. Subgroup analysis of associations between oral health status and colorectal cancer incidence and mortality

| **Subgroup** | **Incidence rate** | | | | **Mortality** | | | |
| --- | --- | --- | --- | --- | --- | --- | --- | --- |
|  | **HR^a^** | **LL** | **UL** | **P for  interaction** | **HR^a^** | **LL** | **UL** | **P for interaction** |
| **Age (year)** |  |  |  | 0.185 |  |  |  | 0.216 |
| **< 50** | 1.05 | 0.77 | 1.41 |  | 1.42 | 0.81 | 2.48 |  |
| **≥ 50** | 0.82 | 0.71 | 0.94 |  | 0.88 | 0.71 | 1.09 |  |
| **Sex** |  |  |  | 0.774 |  |  |  | 0.638 |
| **Men** | 0.81 | 0.68 | 0.97 |  | 0.86 | 0.64 | 1.15 |  |
| **Women** | 0.84 | 0.71 | 1.00 |  | 0.95 | 0.71 | 1.26 |  |
| **Menopause status** |  |  |  | 0.363 |  |  |  | 0.810 |
| **Premenopausal** | 0.90 | 0.59 | 1.37 |  | 1.33 | 0.58 | 3.03 |  |
| **Perimenopausal** | 2.16 | 0.98 | 4.76 |  | 1.24 | 0.15 | 9.88 |  |
| **Postmenopausal** | 0.82 | 0.67 | 0.99 |  | 0.92 | 0.67 | 1.25 |  |
| **Baseline BMI (kg/m^2^)** |  |  |  | 0.171 |  |  |  | 0.446 |
| **< 24** | 0.89 | 0.76 | 1.05 |  | 0.97 | 0.75 | 1.26 |  |
| **24-<28** | 0.69 | 0.55 | 0.87 |  | 0.73 | 0.48 | 1.09 |  |
| **≥ 28** | 0.88 | 0.63 | 1.24 |  | 1.03 | 0.57 | 1.86 |  |
| **Study site** |  |  |  | 0.281 |  |  |  | 0.527 |
| **Urban** | 0.87 | 0.73 | 1.05 |  | 0.92 | 0.65 | 1.29 |  |
| **Rural** | 0.76 | 0.65 | 0.89 |  | 0.80 | 0.63 | 1.01 |  |
| **Educational levels** |  |  |  | 0.438 |  |  |  | 0.868 |
| **No formal school** | 0.77 | 0.61 | 0.97 |  | 0.99 | 0.71 | 1.38 |  |
| **Primary or middle school** | 0.94 | 0.76 | 1.17 |  | 0.89 | 0.62 | 1.28 |  |
| **High school and above** | 0.80 | 0.65 | 0.99 |  | 0.87 | 0.59 | 1.26 |  |
| **Marital status** |  |  |  | 0.251 |  |  |  | 0.337 |
| **Currently married** | 0.80 | 0.70 | 0.92 |  | 0.84 | 0.67 | 1.06 |  |
| **Other** | 0.99 | 0.73 | 1.34 |  | 1.11 | 0.71 | 1.74 |  |
| **Household income, ¥ per year** |  |  |  | 0.286 |  |  |  | 0.449 |
| **< ¥10,000** | 0.72 | 0.57 | 0.90 |  | 0.74 | 0.53 | 1.03 |  |
| **¥10,000–19,999** | 0.90 | 0.72 | 1.13 |  | 1.14 | 0.79 | 1.66 |  |
| **¥20,000–34,999** | 0.75 | 0.57 | 0.99 |  | 0.85 | 0.53 | 1.37 |  |
| **≥ ¥35,000** | 1.00 | 0.75 | 1.34 |  | 0.98 | 0.56 | 1.69 |  |
| **Physical activity (MET hr/day)** |  |  |  | 0.569 |  |  |  | 0.349 |
| **< 12.29** | 0.87 | 0.72 | 1.04 |  | 1.02 | 0.76 | 1.36 |  |
| **12.29-< 25.3** | 0.83 | 0.66 | 1.03 |  | 0.72 | 0.49 | 1.06 |  |
| **≥ 25.3** | 0.73 | 0.55 | 0.96 |  | 0.93 | 0.61 | 1.42 |  |
| **Smoking status** |  |  |  | 0.633 |  |  |  | 0.834 |
| **Never smoker** | 0.85 | 0.72 | 1.00 |  | 0.92 | 0.69 | 1.23 |  |
| **Current or ever smoker** | 0.80 | 0.66 | 0.96 |  | 0.88 | 0.66 | 1.18 |  |
| **Alcohol drinking** |  |  |  | 0.925 |  |  |  | 0.471 |
| **Non-drinker** | 0.83 | 0.69 | 1.01 |  | 0.98 | 0.73 | 1.32 |  |
| **Current or ever drinker** | 0.82 | 0.69 | 0.96 |  | 0.84 | 0.63 | 1.11 |  |
| **Aspirin use** |  |  |  | 0.305 |  |  |  | 0.293 |
| **Yes** | 0.54 | 0.19 | 1.50 |  | 0.26 | 0.03 | 2.33 |  |
| **No** | 0.91 | 0.68 | 1.21 |  | 0.93 | 0.56 | 1.54 |  |
| **History of diabetes** |  |  |  | 0.749 |  |  |  | 0.654 |
| **Yes** | 0.88 | 0.59 | 1.29 |  | 0.78 | 0.39 | 1.55 |  |
| **No** | 0.82 | 0.72 | 0.94 |  | 0.92 | 0.74 | 1.13 |  |
| **Family history of diabetes** |  |  |  | 0.659 |  |  |  | 0.804 |
| **Yes** | 0.87 | 0.66 | 1.14 |  | 0.98 | 0.60 | 1.62 |  |
| **No** | 0.81 | 0.70 | 0.93 |  | 0.91 | 0.72 | 1.14 |  |

**^a^** Adjusted for age (continuous), sex (male, female), body mass index (BMI, continuous), study sites (10 sites), education level (no formal school, primary or middle school, high school and above), marital status (married, other), household income per year (< ¥10,000, ¥10,000–19,999, ¥20,000–34,999, or ≥ ¥35,000), alcohol consumption (non-drinker, occasional drinker, former drinker, or regular drinker), smoking status (never smoker, occasional smoker, former smoker, or regular smoker), physical activity in metabolic equivalent tasks (MET) hours a day (continuous), aspirin prescription for CVD (no, yes, or missing), menopausal status (pre-menopausal or post-menopausal, women only), personal history of diabetes (no, yes), and family history of cancer (no, yes).
